# Supplementary material for: Causal Relationship between Aging and Anorexia Nervosa: A White-Matter-Microstructure-Mediated Mendelian Randomization Analysis
Source: Biomedicines. 2024 Aug 16;12(8):1874. doi: 10.3390/biomedicines12081874 (PMC11351342; doi:10.3390/biomedicines12081874)
Supplement: Supplementary file 1 [file biomedicines-12-01874-s001.zip › Supplementary Figure/Figure S4/Singe SNP plot of MR analysis for TL to ubm-b-1623.pdf]

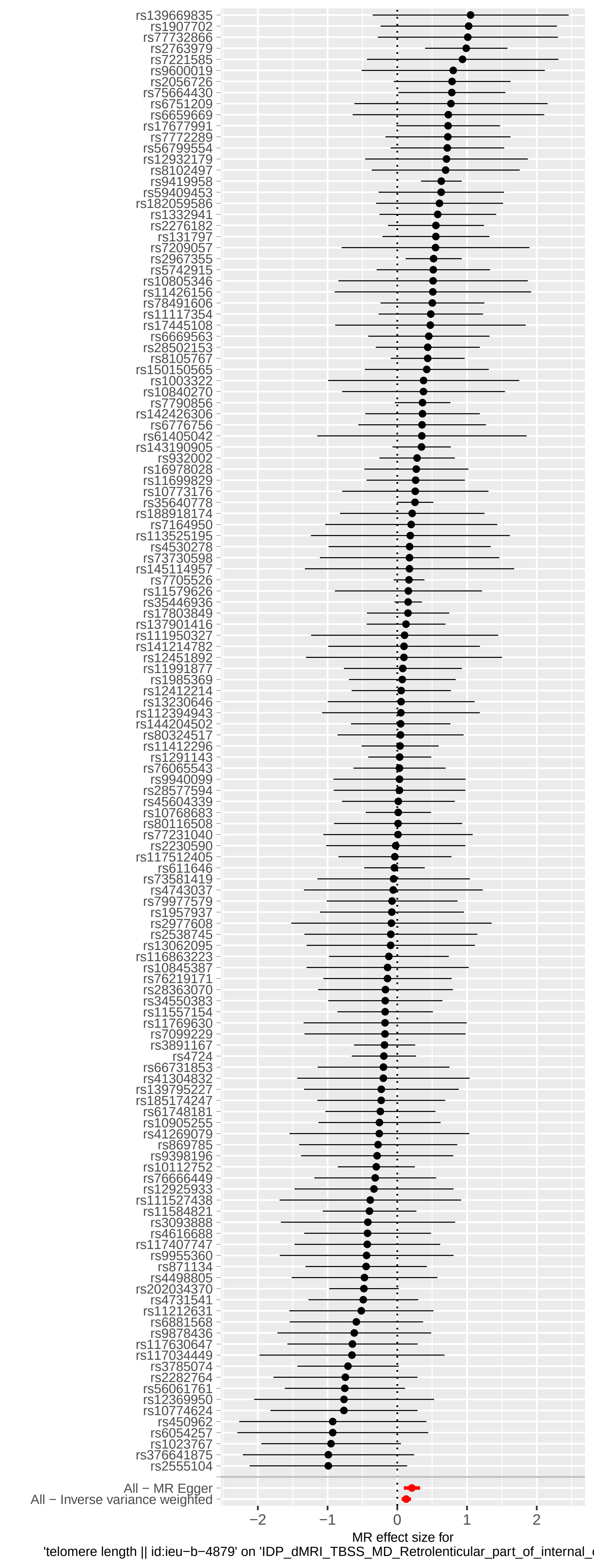
rs139669835
rs1907702
rs77732866
rs2763979
rs7221585
rs9600019
rs2056726
rs75664430
rs6751209
rs6659669
rs17677991
rs7772289
rs56799554
rs12932179
rs8102497
rs9419958
rs59409453
rs182059586
rs1332941
rs2276182
rs131797
rs7209057
rs2967355
rs5742915
rs10805346
rs11426156
rs78491606
rs11117354
rs17445108
rs6669563
rs28502153
rs8105767
rs150150565
rs1003322
rs10840270
rs7790856
rs142426306
rs6776756
rs61405042
rs143190905
rs932002
rs16978028
rs11699829
rs10773176
rs35640778
rs188918174
rs7164950
rs113525195
rs4530278
rs73730598
rs145114957
rs7705526
rs11579626
rs35446936
rs17803849
rs137901416
rs111950327
rs141214782
rs12451892
rs11991877
rs1985369
rs12412214
rs13230646
rs112394943
rs144204502
rs80324517
rs11412296
rs1291143
rs76065543
rs9940099
rs28577594
rs45604339
rs10768683
rs80116508
rs77231040
rs2230590
rs117512405
rs611646
rs73581419
rs4743037
rs79977579
rs1957937
rs2977608
rs2538745
rs13062095
rs116863223
rs10845387
rs76219171
rs28363070
rs34550383
rs11557154
rs11769630
rs7099229
rs3891167
rs4724
rs66731853
rs41304832
rs139795227
rs185174247
rs61748181
rs10905255
rs41269079
rs869785
rs9398196
rs10112752
rs76666449
rs12925933
rs111527438
rs11584821
rs3093888
rs4616688
rs117407747
rs9955360
rs871134
rs4498805
rs202034370
rs4731541
rs11212631
rs6881568
rs9878436
rs117630647
rs117034449
rs3785074
rs2282764
rs56061761
rs12369950
rs10774624
rs450962
rs6054257
rs1023767
rs376641875
rs2555104
All − MR Egger
All − Inverse variance weighted
−2
−1
0
1
2
MR effect size for
'telomere length || id:ieu−b−4879' on 'IDP_dMRI_TBSS_MD_Retrolenticular_part_of_internal_c
